# Investigation of the immunogenicity of Zika glycan loop

**DOI:** 10.1186/s12985-020-01313-1

**Published:** 2020-03-31

**Authors:** Elizabeth A. Henderson, Christina C. Tam, Luisa W. Cheng, Annie Elong Ngono, Anh-Viet Nguyen, Sujan Shresta, Matt McGee, Hal Padgett, Laurence K. Grill, Mikhail Martchenko Shilman

**Affiliations:** 1 Henry E. Riggs School of Applied Life Sciences, Keck Graduate Institute, Claremont, CA 91711 USA; 2 Foodborne Toxin Detection and Prevention Research Unit, Western Regional Research Center, United States Department of Agriculture (USDA), Albany, CA 94710 USA; 3 Division of Inflammation Biology, La Jolla Institute for Allergy and Immunology, La Jolla, CA 92037 USA; 4 Novici Biotech LLC, Vacaville, CA 95688 USA

**Keywords:** Zika, Envelope, Glycan loop, Neutralizing antibodies

## Abstract

**Background:**

Zika virus (ZIKV) is a major human pathogen and member of the *Flavivirus* genus. Previous studies have identified neutralizing antibodies from Zika patients that bind to quaternary epitopes across neighboring envelope (E) proteins, called E dimer epitopes (EDE). An asparagine-linked glycan on the “glycan loop” (GL) of the ZIKV envelope protein protects the functionally important “fusion loop” on the opposite E subunit in the dimer, and EDE antibodies have been shown to bind to both of these loops. Human EDE antibodies have been divided into two subclasses based on how they bind to the glycan loop region: EDE1 antibodies do not require glycosylation for binding, while EDE2 antibodies strongly rely on the glycan for binding.

**Methods:**

ZIKV GL was expressed on tobacco mosaic virus nanoparticles. Mice were immunized with GL or full-length monomeric E and the immune response was analyzed by testing the ability of sera and monoclonal antibodies to bind to GL and to neutralize ZIKV in in vitro cellular assay.

**Results:**

We report here the existence of ZIKV moderately neutralizing antibodies that bind to E monomers through epitopes that include the glycan loop. We show that sera from human Zika patients contain antibodies capable of binding to the unglycosylated glycan loop in the absence of the rest of the envelope protein. Furthermore, mice were inoculated with recombinant E monomers and produced neutralizing antibodies that either recognize unglycosylated glycan loop or require glycan for their binding to monomeric E. We demonstrate that both types of antibodies neutralize ZIKV to some extent in a cellular virus neutralization assay.

**Conclusions:**

Analogous to the existing EDE antibody nomenclature, we propose a new classification for antibodies that bind to E monomer epitopes (EME): EME1 and EME2 for those that do not require and those that do require glycan for binding to E, respectively.

## Background

Zika virus (ZIKV) belongs to the *Flavivirus* genus, which is composed of such viruses as yellow fever virus (YFV) and dengue virus (DENV). *Aedes* genus mosquitoes transmit ZIKV to humans [1], but it can also be transmitted from one human to another perinatally, sexually, and through breast milk and blood transfusions [1]. The World Health Organization declared ZIKV a Public Health Emergency of International Concern in 2016 [2]. Recent outbreaks have shown that ZIKV infections are associated with a variety of severe outcomes, such as Guillain–Barré syndrome [3] and congenital Zika syndrome [4].

Structurally, ZIKV includes three structural proteins that come from a single precursor polyprotein: the capsid protein (C), the envelope protein (E), and the membrane protein (M) [5–8]. The C forms an inner shell around the viral RNA, and 180 copies of E/M anchored in the lipid membrane form an icosahedral outer shell. In mature virions, 90 E-M heterodimers form a smooth surface in which the E homodimers lie parallel to each other in a herringbone pattern. The E is antigenically important and has been shown to induce protective immunity. However, there is a high level of conservation among the flavivirus genus Es, and cryo-electron microscopy studies have shown that the overall structural architecture of mature ZIKV and DENV particles is very similar [5, 6]. Indeed, all flavivirus Es are antigenically related, which can lead to cross-protection or antibody-dependent enhancement of disease.

**Fig. 1:**
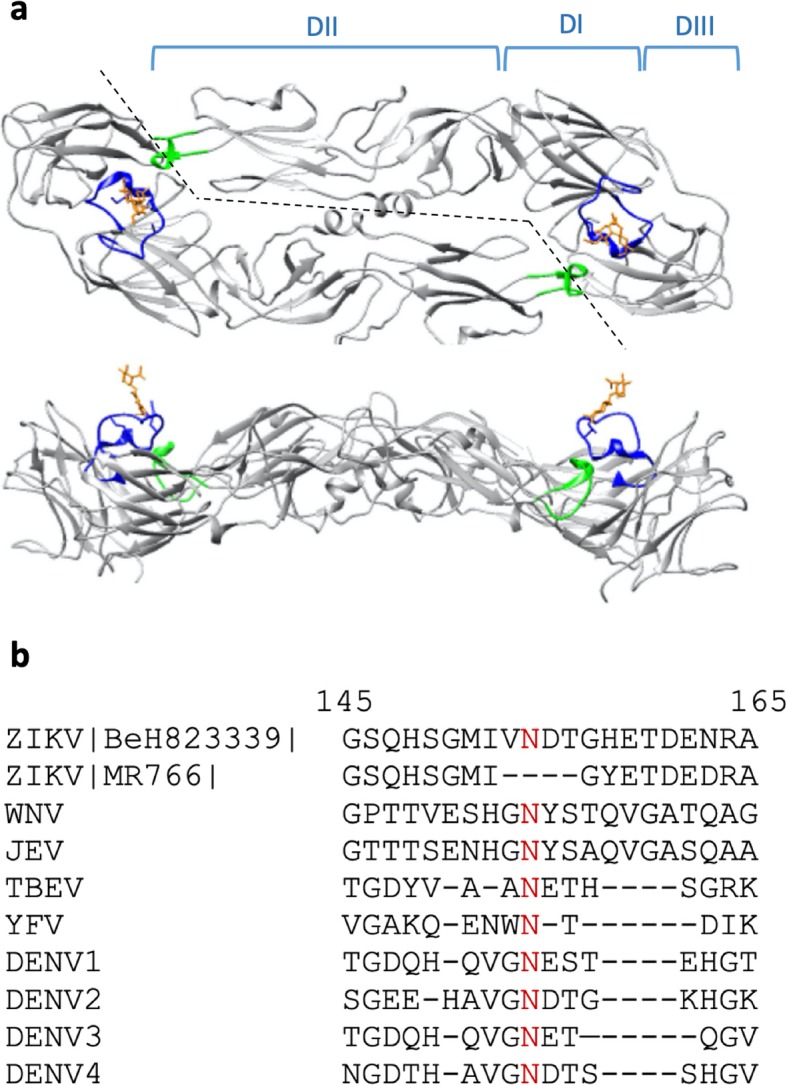
Sequence alignment and immunoreactivity of GL. **a** Top and side views of the Zika envelope protein dimer (Protein Data Bank structure 5IRE) with domains I, II, and III (DI, DII, DIII) indicated above the structure in the top panel. The glycan loop and the glycan are indicated in blue and orange, respectively, and the fusion loop is indicated green. **b** GL sequence alignment of Asian-lineage strain of ZIKV BeH823339, the prototype Ugandan MR766 strain, and other flaviviruses. The numbers indicate the position of the amino acids in the ZIKV envelope protein reference sequence, and the red N indicates the conserved glycosylation site

The E consists of three structural domains (DI, DII, DIII), an α-helical stem, and an α-helical transmembrane region [6] (Fig. 1a). The E mediates binding to host cell receptors and entry into host cells and is the target of many neutralizing antibodies (NAbs) [9–14]. Analyses of sera from DENV and ZIKV-infected donors have shown that the most potent ZIKV-neutralizing monoclonal antibodies (mAbs) are directed against E epitopes [11, 12, 15], with the most potent neutralizing antibodies usually recognizing tertiary epitopes or quaternary epitopes [8, 10, 11, 15–18]. Tertiary epitopes are those contained within a single E monomer, and neutralizing tertiary E epitopes include areas like the lateral ridge of DIII [10, 11, 15, 16] and the DI-DII hinge [10]. In addition, several quaternary epitope-binding mAbs isolated from DENV patients are capable of cross-neutralizing ZIKV and have been divided into two categories, EDE1 and EDE2 (E dimer epitopes). EDE1 and 2 antibodies stabilize the dimeric state of the E and have very poor binding to the E monomer [8, 19]. EDE1 antibodies also bind better to unglycosylated strains of ZIKV, such as the original 1947 MR766 Uganda strain, while EDE2 antibodies bind better to glycosylated strains of ZIKV but show less potent neutralization of ZIKV [8].

One significant difference between the envelope proteins of DENV and ZIKV lies in the glycan loop (GL) region (Fig. 1b). In the ZIKV E, this loop contains an additional 6–9 amino acids and protrudes further from the surface of one E monomer to partially cover the FL of the adjacent E monomer [5, 6, 20]. It has been recently shown that the glycosylation of N154 contributes to the pathogenicity and infectivity of ZIKV in mammals [21–23] and mosquitos [24]. Hypotheses on the function of the extended GL on ZIKV E include modulation of access to the FL [5], attachment to cellular receptors [5], and regulation of antigenicity via changes in E conformation [20].

Identifying NAbs and their epitopes is an essential part of understanding the protective humoral immune response. Since monomeric E tends to elicit predominantly non-neutralizing fusion loop antibodies, it may be a less effective vaccine antigen than dimerized E [25]. However, the resulting immune response could yield novel antibodies against E that are not identified when using dimerized antigen. Future efforts that include vaccinations with monomeric E would likely exclude the development of EDE type antibodies and would rely on NAbs that have recognition sites on an E monomer. These antibodies could either rely on the N154 glycan for binding or not. In this study, we sought to explore the immune response to E GL unique to ZIKV. The discovery of a new class of mAbs may have significant implications for the development of ZIKV vaccines, therapeutics, and diagnostics with analogous significance to other flaviviruses.

## Methods

In this study we used various rE constructs which are summarized in Table 1.

**Table 1:** Details of ZIKV rE constructs used in this study. (−) indicates the lack of glycosylation

ZIKV E Construct name	E residues	ZIKV strain	Expression host	Glycosylated (Y/N)
TMV-GL	145–165	BeH823339 - Asian	*Nicotiana benthamiana*	N
rE(−)	1–408	BeH815744 - Asian	*Escherichia coli*	N
rE	1–408	BeH823339 - Asian	*Nicotiana plumbaginifolia*	Y
TMV-E	1–408	BeH823339 - Asian	*Nicotiana benthamiana*	Y

### Viral nanoparticle expression and purification

The gene for the TMV coat protein (CP) was genetically fused at the C-terminus with the ZIKV E GL (aa 145–165, GenBank accession no. AMK49164) in a pCR2.1 plasmid. The plasmid was used as a template for PCR-amplification of the CP-epitope sequence and then cloned into pJLTRBO [26]. The recombinant pJLTRBO plasmid was transformed into chemically competent *Agrobacterium tumefaciens* strain GV3101, followed by expansion and vacuum infiltration of *Nicotiana benthamiana* plants as indicated in [27] with the following exceptions: *Agrobacterium* induction media (10 mM MES, 10 mM MgCl_2_, 150 μM acetosyringone), and a 75 kPA vacuum was applied for 5 min. The virus was extracted from leaf tissue as described in [28] with slight modifications to the extraction buffer composition (50 mM sodium acetate, 0.1% sodium metabisulfite (w/v), 0.01% BME (v/v), pH 5.0) and omission of the chloroform extraction. The viral nanoparticles were further purified using sucrose density gradient centrifugation as described in [29].

The ZIKV E (amino acids 1–408, GenBank accession no. AMK49164.2, which corresponds to strain BeH823339 of the Asian lineage) with an N-terminal extensin secretory signal peptide from *Nicotiana plumbaginifolia,* a C-terminal InaD Pdz domain (amino acids 2–98 GenBank accession no. 1IHJ_A, cys53ala mutation), and 6xHis purification tag were codon-optimized for expression in *Nicotiana* and cloned into a tobamovirus iBioLaunch vector (iBio Inc.). ZIKV-E with the extensin secretory signal peptide and a C-terminal 6xHis purification tag and KDEL was also cloned into an iBioLaunch vector. 5- or 6-days post-infiltration, plants were ground in 50 mM CAPS (pH 10.9), 500 mM NaCl, 200 mM sucrose, 40 mM ascorbic acid, 2 mM PMSF. The lysate was filtered through spun-bonded polypropylene fabric, centrifuged at 30,100 x g, acidified with 1 M NaOAc (pH 4.5), centrifuged, neutralized with 1 M Tris (pH 9.5), centrifuged, and filtered at 0.2 μm. The recombinant protein was then purified from the lysate by IMAC with Nickel-NTA agarose (Qiagen) or using a 1 mL HisTrap™ HP (GE Healthcare), dialyzed into PBS, and concentrated with a 10 kDa cutoff spin concentrator. rE was produced and purified as described above for rE-InaD, except that it did not contain the C-terminal InaD Pdz domain.

The TMV-EFCA dock-and-lock virus scaffold was cloned by the addition of the amino acid sequence “EFCA” from the NorpA protein to the C-terminus of the TMV capsid protein in a TMV vector [30]. RNA was transcribed using the Ambion mMESSAGE mMACHINE kit (Invitrogen) and encapsidated with purified TMV U1 capsid protein [31]. The virus was then rub-inoculated onto *N. benthamiana* plants by manual abrasion with diatomaceous earth. Leaf tissue was harvested about 12 days later, and the virus was purified by PEG precipitation [32]. rE-TMV viral-like particles (VLP) were prepared using the “Dock-and-Lock” method [33, 34] to attach the rE-InaD fusion protein to TMV-EFCA virus scaffold covalently. The VLP was then separated from unbound rE-InaD by precipitation of the VLP with 0.5 M NaCl and 4% PEG. The supernatant was removed, and the VLP resuspended in PBS. Docking confirmation and rE aggregation were evaluated by Coomassie-stained SDS-PAGE gels of both reduced and non-reduced samples. Proteins were heated in 1X Laemmli buffer (with either no reducing agent or 1.8% β-mercaptoethanol) at 95 degrees for 5 min. Samples were then run on 4–20% SDS-PAGE gel, stained with Coomassie G-250, and imaged with a BioRad GS-800 Calibrated Densitometer.

### Western blot

For Western blots, recombinant ZIKV E protein (Gene accession # AMA12087) (eEnzyme catalog # ZV-E-005P) was used. SDS-PAGE gels were transferred onto 0.2 μm nitrocellulose membrane (BioRad) for 90 mins at 150 mAmps. The membrane was blocked with 5% (w/v) nonfat dry milk in TBS at room temperature for 1 h then incubated with rabbit anti-ZIKV envelope protein (strain BeH815744) (eEnzyme catalog # ZV-E-0105), anti-DENV2 E (Fisher Scientific catalog # PA5–32246), or anti-ZIKV envelope protein (strain Mr766) (Abiocode catalog # R3771–8) polyclonal antibodies (1:2000 dilution) in 5% (w/v) milk/TBS overnight at 4 °C. After washing with TBS and TBST, the membrane was incubated with goat anti-rabbit HRP-conjugated secondary antibodies (BioRad) (1:3000 dilution) for 1.5 h at room temperature. After washing with TBS and TBST, a chemiluminescent signal was generated by incubating the membrane for 5 min with Clarity Western ECL Substrate (BioRad) and was documented using the Azure c500 (Azure Biosystems).

### Dot blot and ELISA analysis

TMV-GL, unglycosylated rE (eEnzyme, catalog # ZV-E-005P), plant-glycosylated rE, Dengue Envelope-2 (DENV) (ProSpec catalog # DEN-022), or tick-borne encephalitis virus (TBEV) rE (ProSpec catalog # TBE-283) were diluted in TBS spotted onto nitrocellulose membrane and allowed to air-dry. The membranes were blocked for 1 h in 10% NF milk in TBS, followed by incubation with the anti-E Z3L1 mAb (Absolute Antibody catalog # Ab00940–10.0) diluted 1:1000 or with a 1:500 dilution of human serum in blocking buffer and incubated overnight at 4 °C. The membranes were washed three times with TBST, followed by one wash with TBS. The blots were incubated for 1 h at room temperature with 1:2000 dilution of secondary antibody (goat anti-human IgG – peroxidase (Sigma)) followed by the addition of Clarity Western ECL Substrate (BioRad) and visualization of chemiluminescence using an Azure Biosystems C500 imager with an exposure time of 5 min. *ImageJ* software was used to digitally quantify the mean inverted pixels of the signal and corrected by that of the background. Normalized fold changes were subsequently calculated by dividing the corrected mean pixels of the signal of TMV-GL by that of TMV.

The binding of human mAb Z3L1 to unglycosylated GL, plant-glycosylated rE, and unglycosylated rE (eEnzyme, catalog # ZV-E-005P) was analyzed by ELISA in triplicate wells. Moreover, mouse sera were analyzed for glycan loop-specific antibody responses by ELISA. Briefly, 96-well plates were coated with protein antigens diluted to 10 ng/μL in carbonate buffer, incubated overnight at 4 °C, and then blocked with 5% non-fat milk in TBS for 1 h at room temperature. Z3L1 diluted to 10 ng/μL in blocking buffer was added to the plates and then incubated at room temperature for 2 h. In other experiments, serially diluted mouse sera were added to the plates and then incubated overnight at 4 °C. Following two washes with PBST and one wash with PBS, the plates were incubated with a 1:2000 dilution of secondary antibody (goat anti-human or goat anti-mouse IgG – peroxidase (Sigma)) for 1 h at room temperature then washed as above. OPD substrate (Sigma) was added, and the absorbance at 450 nm was measured on a SpectraMax Plus plate reader (Molecular Devices). The background was measured as above, except the blocking buffer was used instead of antigen. For the unglycosylated rE antigen, anti-ZIKV rE (eEnzyme catalog # ZV-E-0105) was used as a positive control with a goat anti-rabbit HRP-conjugated secondary antibody (BioRad). The antibody endpoint titers were defined as the reciprocal of the highest serum dilution that gave a reading above the cutoff. Cutoff values were determined for each dilution of adjuvant only serum as described in [35].

### Immunization of mice and generation of hybridomas

SP2/0 mouse myeloma cells and all hybridoma cells were maintained in hybridoma medium (HM), consisting of IMDM (Sigma #I-7633) with 10% fetal calf serum (cHM). Hybridomas were selected following cell fusion using HAT selection medium. The macrophage conditioned medium (MϕCM) was prepared as described in [36]. A mixture of 40% cHM and MϕCM was used for all cell-cloning procedures [37].

Five groups of three-month-old female BALB/c mice (three mice per group) (Simonsen Labs) were intraperitoneally immunized with 25 μg of antigen: TMV-GL, TMV-E, rE, or a control (TMV or sterile PBS) and adjuvant (Sigma Adjuvant System). Boosters were given approximately every 3 weeks for 4 rounds. Sera were obtained and evaluated for reactivity to the various target antigen using a direct-binding ELISA format. 96-well plates were coated with antigens at 30 μL/well of a 1.0 μg/mL in carbonate buffer overnight at 4 °C, and then blocked with 3% non-fat dry milk in TBST for 1 h at 37 °C. The plates were then washed 3x with TBST. Next, sera were added (30 μL/well) and the plates were incubated at 37 °C for 1 h, washed 3x, and 30 μL/well of a 1/5000 dilution of peroxidase-conjugated goat anti-mouse sera (Sigma) was added for 1 h at 37 °C. The plates were then washed 3x with TBST. The luminescence substrate (SuperSignal West Pico chemiluminescence) was added according to the manufacturer’s recommendation, and luminescent counts recorded using the Victor 3 (Perkin Elmer). All protocols were approved by the Western Regional Research Center Institutional Animal Care and Use Committee (Protocol # 16–1).

Mice that were positive for reactivity to antigen were immunized once more 3 days before the start of cell fusion. Mice were euthanized, and their splenocytes were fused with SP2/0 myeloma cells using polyethylene glycol as previously described in [38]. Following cell fusion, the cells were suspended in 100 mL of HAT selection medium supplemented with 10% fetal calf serum and 10% MϕCM, dispensed into ten, 96-well tissue culture plates, and incubated for 10 to 14 days at 37 °C in 5% CO_2_ before screening for antibody production.

Supernatants from cell fusion plates were screened using a direct-binding ELISA. Clear Nunc Maxisorp microtiter plates were rinsed with distilled water and coated by incubating 100 μL/well of a 1.0 μg/mL rE in sodium carbonate buffer overnight at 4 °C. The ELISA plates were processed as described above and the signal was detected by adding 150 μL/well of K-Blue® enhanced substrate (TMB) (Neogen) to each of the 96-well plates until color development was sufficient. The signal was read at 650 nm on the VersaMax (Molecular Dynamics). Cells from the wells giving positive signals for antibody production were cloned by limiting dilution (3x) with screening using a direct ELISA [37].

### Flow cytometry-based ZIKV neutralization assay

A flow cytometry-based assay was used to measure the capacity of the mouse sera and the hybridoma supernatants to neutralize ZIKV (strain SD001) in U937 + DC-SIGN cells as previously described in [39]. Briefly, sera were heat-inactivated for 30 min at 56 °C and then serially diluted in RPMI medium (containing 1% penicillin/streptomycin and 1% HEPES) on a 96-well flat-bottom plate. A sufficient amount of ZIKV SD001 causing 4.5–20% of infection in U937 DC-SIGN, determined via titration of the virus, was added to the sera or hybridoma supernatants (mAbs) and incubated for 1 h at 37 °C. The inoculum containing the serum or mAbs with the virus, was added to a 96-well round-bottom plate containing 1.0 × 10^5^ U937 DC-SIGN cells per well and incubated for 2 h at 37 °C with infection media without FBS. Cells and virus incubated in the absence of serum/mAbs served as the positive control. Plates were then centrifuged at 1500 rpm for 3 min at 4 °C, the supernatants were aspirated, fresh media with 10% FBS was added, and the cells were incubated for 20 h at 37 °C. Finally, cells were harvested, stained with PE-conjugated anti-CD209 (DC-SIGN clone DNC246), incubated with Cytofix/Cytoperm solution (BD Biosciences), and stained intracellularly with FITC-conjugated 4G2 (to E). The cells were analyzed on a FACSCanto II flow cytometer (BD Biosciences) and the percentage of infected cells was determined using FlowJo software (Tree Star) as [(% infected cells in the absence of serum/supernatant - % infected cells in the presence of serum/supernatant)/(% infected cells in the absence of serum/supernatant) × 100%].

### Ethical approval

The animal work was conducted at the Small Animal Facility of the Western Regional Research Center, U.S. Department of Agriculture in Albany, California. Animal work was approved by the Animal Care and Use Committee of the Albany USDA. The Animal Welfare Assurance Approval is D16–00811 (A4589–01).

## Results

### Expression of unglycosylated ZIKV GL on tobacco mosaic virus nanoparticles

In order to investigate whether antibodies exist that are capable of binding exclusively to GL, we expressed ZIKV E aa 145–165 on the surface of the Tobacco Mosaic Virus (TMV). TMV is a rod-shaped virus comprised of an RNA genome encapsidated by 2130 coat protein monomers, which is optimal for uptake by antigen-presenting cells and subsequent CD4+ T cell priming and also for potent cross-linking of B-cell receptors [40]. Expressing the GL epitope on the C-terminus of the TMV coat protein allowed us to create TMV nanoparticles that display GL in a repetitive and highly ordered manner, which facilitates an enhanced immune response [41]. Immunizing mice with TMV-GL allowed us to study the immune response to GL in the absence of the rest of the E. TMV-GL was also utilized in subsequent immunoassays to assess antibody binding to the GL region. Moreover, Barba-Spaeth et al. showed that mAbs capable of binding to the GL region in the absence of the N154 glycan are better at neutralizing glycosylated strains of ZIKV [8], and because TMV-GL is assembled in the cytoplasm of *Nicotiana benthamiana* cells, neither TMV nor GL are glycosylated [42, 43]. Thus, our TMV-GL allowed us to look for mAbs that bind to GL independently of the N154 glycan.

**Fig. 2:**
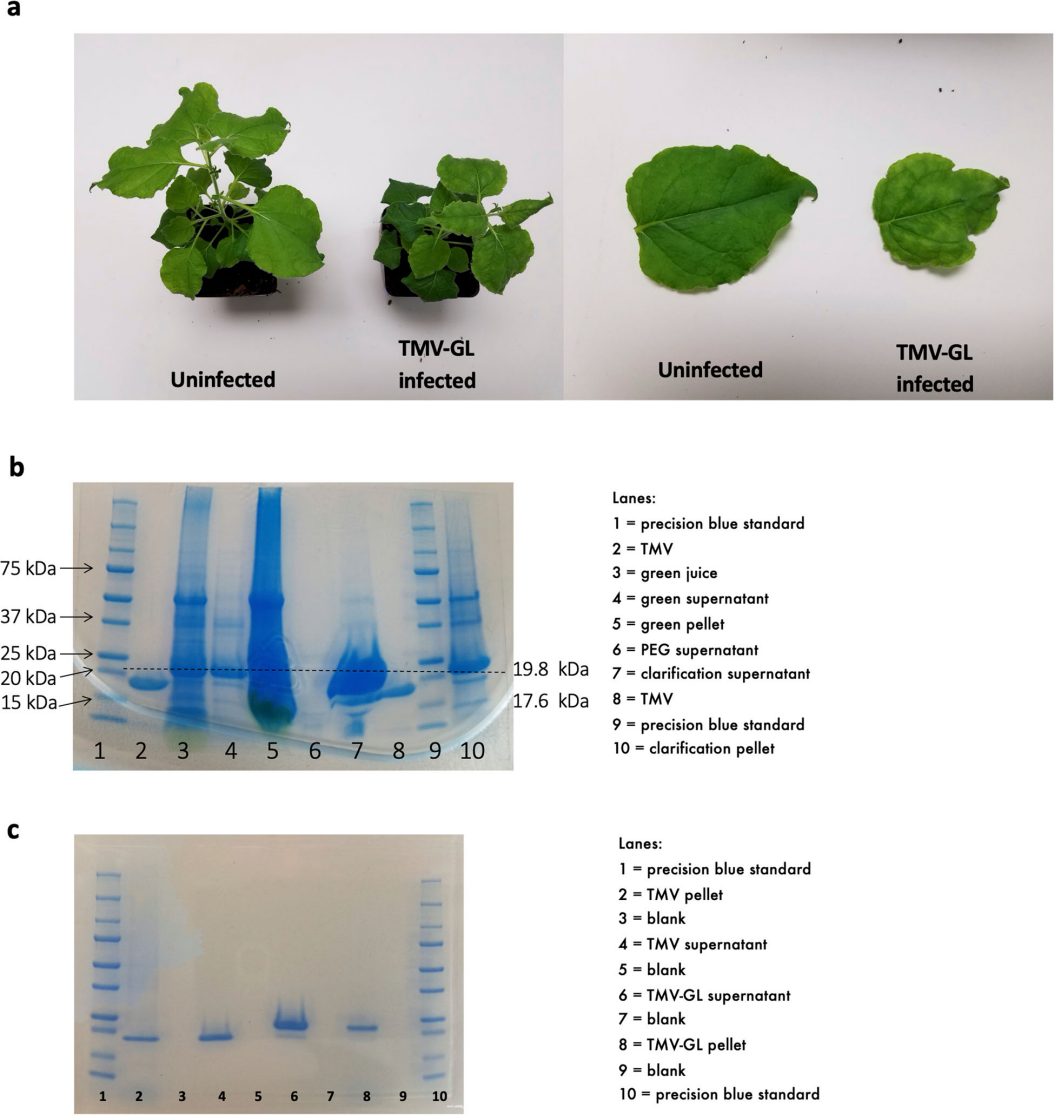
TMV-GL expression in *Nicotiana benthamiana*. **a** An uninfected *N. benthamiana* plant and leaf (left) compared to a TMV-GL infected plant and leaf (right). **b** TMV-GL was extracted from leaf tissue using PEG precipitation, and fractions were analyzed via SDS-PAGE. c) TMV-GL and TMV were purified using sucrose gradient ultracentrifugation

TMV-GL was extracted from *N. benthamiana* leaf tissue at 14 days post-infection (Fig. 2a) and analyzed on SDS-PAGE. Following PEG-precipitation, the majority of TMV-GL was found in the clarification supernatant (Fig. 2b), and was further purified using sucrose gradient ultracentrifugation (this process was also used to purify the TMV negative control) (Fig. 2c) SDS–PAGE analysis of purified TMV-GL revealed two bands, with the higher molecular weight band at 19.8 kDa corresponding with the expected molecular weight of a TMV coat protein plus the GL and accounting for approximately 87% of the protein. The smaller band at approximately 17.6 kDa corresponds with the estimated molecular weight of the TMV coat protein alone, as shown by the purified TMV. TMV-GL was used in subsequent experiments to screen human sera for GL-binding antibodies and to determine if GL is capable of eliciting a NAbs response in mice.

### Binding characteristics of commercial anti-E antibodies to the unglycosylated GL

**Fig. 3:**
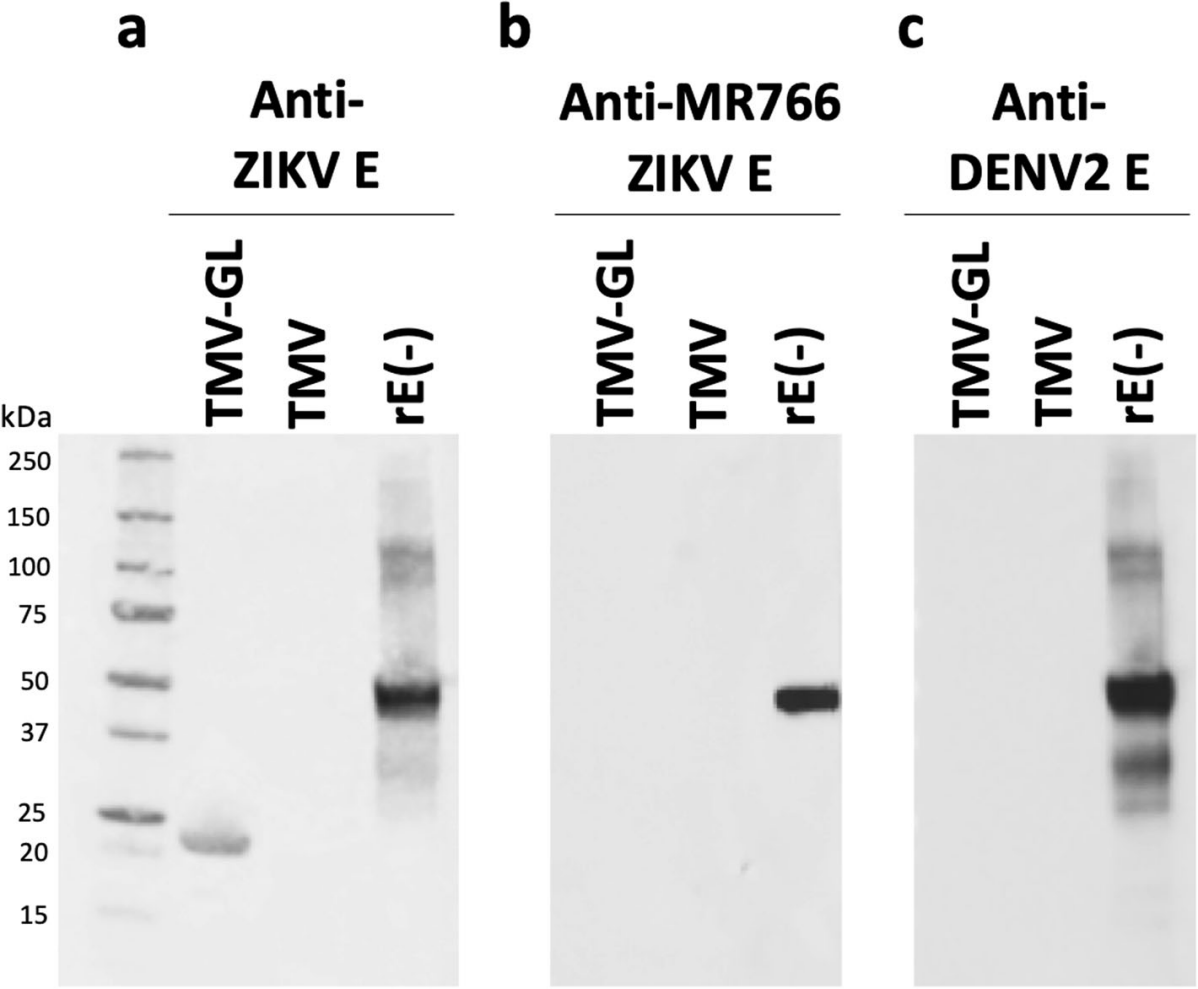
Binding of commercial anti-E antibodies to the unglycosylated GL. PEG-purified TMV-GL, TMV, and ZIKV rE from *E. coli* (rE(−)) were separated on 4–20% SDS-PAGE gel under reducing conditions and then blotted onto a nitrocellulose membrane. The membrane was then incubated with **a** polyclonal anti-ZIKV envelope protein antibody, **b** polyclonal anti-envelope protein antibody from MR766 strain of ZIKV, or c) polyclonal anti-DENV-2 envelope protein antibody. (−) indicates the lack of glycosylation

To investigate whether a commercially available polyclonal Ab raised against E of a Brazilian strain of ZIKV recognized our unglycosylated TMV-GL construct that we intended to use in subsequent animal studies, we performed Western blot analysis. The rE used as a positive control in this experiment was a 55-kDa full-length recombinant E from ZIKV strain BeH815744 expressed in *Escherichia coli*, and, therefore, was also unglycosylated (referred to in this study as rE(−), where the minus indicates the lack of glycosylation (Table 1)). Rabbit polyclonal antibodies raised against a glycosylated ZIKV E bound to TMV-GL and to E but did not bind to the unmodified TMV (Fig. 3a). This data demonstrates that antibodies raised against glycosylated E were able to recognize unglycosylated GL displayed on TMV.

The Uganda MR766 strain of ZIKV does not contain N154 and three of the surrounding aa (i.e., VNDT), and therefore is not glycosylated (Fig. 1b). A rabbit polyclonal Ab raised against aa 140–180 of the MR766 E reacted with the full-length recombinant ZIKV E from strain BeH815744 but did not react with TMV-GL (Fig. 3b). This suggests that antibodies raised against the E of the Uganda MR766 strain, as well as antibodies raised against other strains of ZIKV that lack the VNDT amino acid motif in the GL region, would not recognize the GL region of Es from the majority of ZIKV strains from the Asian lineage, which includes the strains responsible for recent outbreaks.

While there is a relatively high degree of homology between the Es of flaviviruses, there is poor homology in the GL region (Fig. 1b). Thus, we theorized that antibodies raised against other flaviviruses Es would not bind to TMV-GL but would be able to bind to the full-length E. We tested the ability of an anti-DENV-2 E polyclonal antibody raised in rabbits immunized with full-length DENV-2 E for its ability to bind to TMV-GL and the previously-mentioned recombinant ZIKV E. The anti-DENV-2 E antibody bound to ZIKV E (Fig. 3c), which shows that there is indeed significant homology between the DENV-2 and ZIKV Es. The anti-DENV-2 antibody did not bind to TMV-GL, suggesting that the glycan loop region is unique to Zika and does not cross-react with DENV antibodies. This data prompted us to investigate whether ZIKV infection leads to the production of antibodies capable of recognizing unglycosylated GL.

**Fig. 4:**
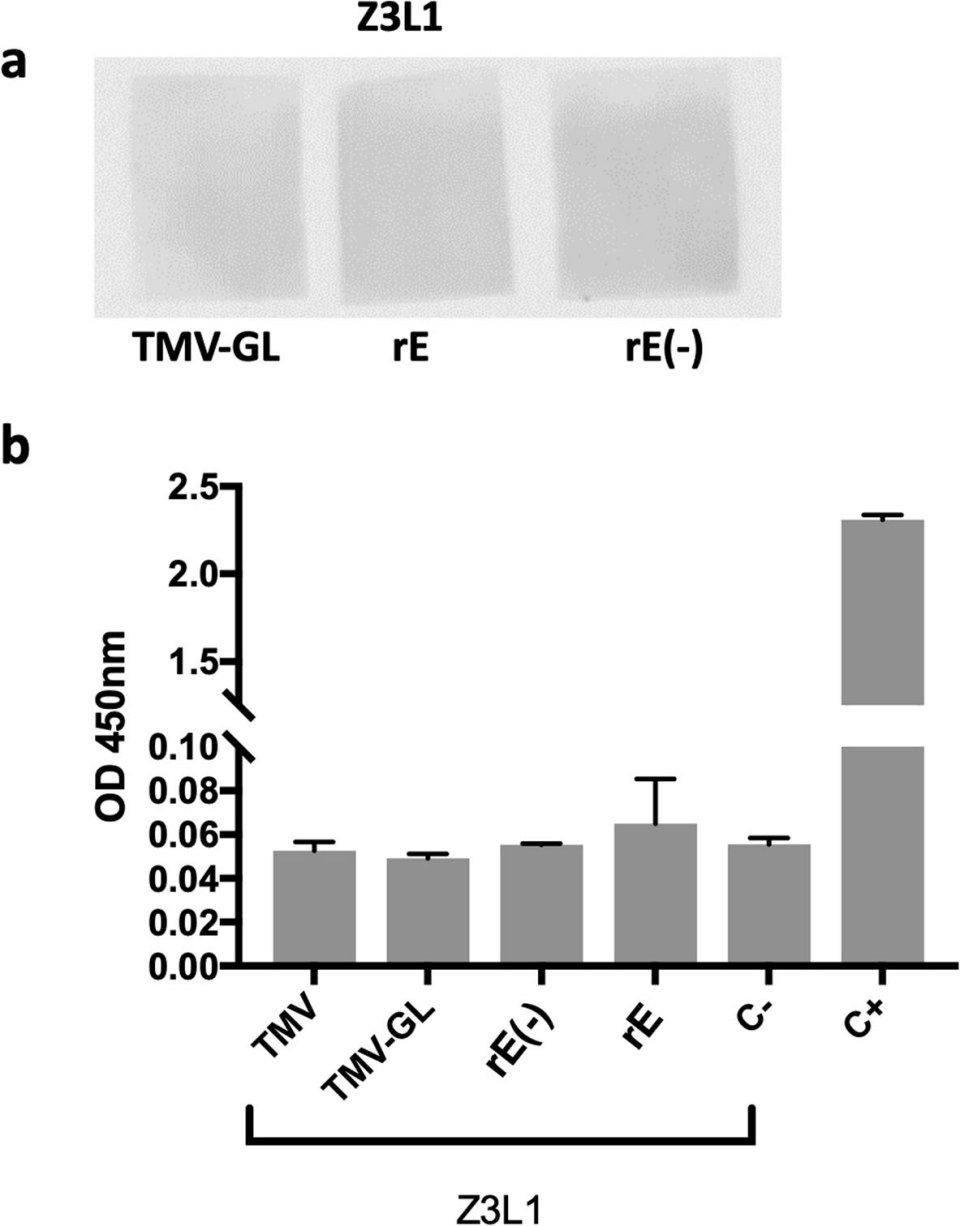
Binding characterization of Z3L1. **a** Binding characterization of Z3L1 by dot blot. TMV-GL, plant-made ZIKV rE, or *E. coli*-made ZIKV rE were dotted onto nitrocellulose, blocked, and then incubated with the human anti-ZIKV mAb Z3L1. A peroxidase-conjugated anti-human IgG secondary antibody and a chemiluminescent substrate were then used to detect any Z3L1 binding. **b** Binding characterization of Z3L1 by ELISA. TMV, TMV-GL, plant-made ZIKV rE (rE) and *E. coli*-made ZIKV rE (rE(−)) were used to coat a 96-well plate. The Z3L1 mAb or an anti-ZIKV rE serum (C+) was added, followed by incubation with HRP-conjugated secondary antibodies and the addition of OPD substrate. The data depicted are the mean absorbance at 450 nm ± SD of triplicate wells. The labels on the x-axis indicate the antigen that was plated. C- indicates that the blocking buffer was used to coat the wells instead of an antigen, thus measuring the background noise from the Z3L1 antibody binding directly to the blocked plate. C+ indicates the use of an anti-ZIKV rE antibody as a positive control with plated *E. coli-*made rE. (−) indicates the lack of glycosylation

To our knowledge, Z3L1 is the only reported ZIKV-neutralizing human antibody with a footprint on monomeric E that includes contacts within GL (i.e.T156, H158, E159) [10]. The binding of Z3L1 to unglycosylated GL displayed on TMV, monomeric unglycosylated rE, and plant-glycosylated rE was analyzed via non-denaturing dot blot and ELISA. Z3L1 did not bind to unglycosylated GL, plant-glycosylated rE, or monomeric unglycosylated rE in the dot blots (Fig. 4a). The ELISA showed similar results: Z3L1 did not bind to unglycosylated GL, plant-glycosylated rE, or monomeric unglycosylated rE to any significant degree above background levels (Fig. 4b), even though Wang et al. showed that Z3L1 does bind weakly to monomeric unglycosylated rE [10]. Thus, the 3 contacts within the GL region do not seem to be sufficient for Z3L1 to bind to unglycosylated GL on TMV.

### IgM and IgG antibodies in sera of human Zika patients react to unglycosylated GL

In a recent study, sera from 51 Zika-infected individuals from Colombia were analyzed for their ability to neutralize ZIKV in vitro [44]. We selected four serum samples from this group of individuals based on the ability of the serum to neutralize 90% of Zika strain H/PF/2013 viral particles at low concentrations (i.e., high reciprocal neutralizing titers) (Table 2).

**Table 2:** Details of serum samples from ZIKV patients

ID	Age	Gender	Days after onset of symptoms	ZIKV H/PF NT_**90**_	Mild symptoms*	Guillain-Barré Syndrome
ARSZ16308	46	F	51	128,080	Yes	No
ARSZGB16015	40	F	14	15,150	Yes	Yes
ARSZGB16031	48	M	30	25,425	Yes	Yes
ARSZ16467	44	F	37	20,211	Yes	No

**Fig. 5:**
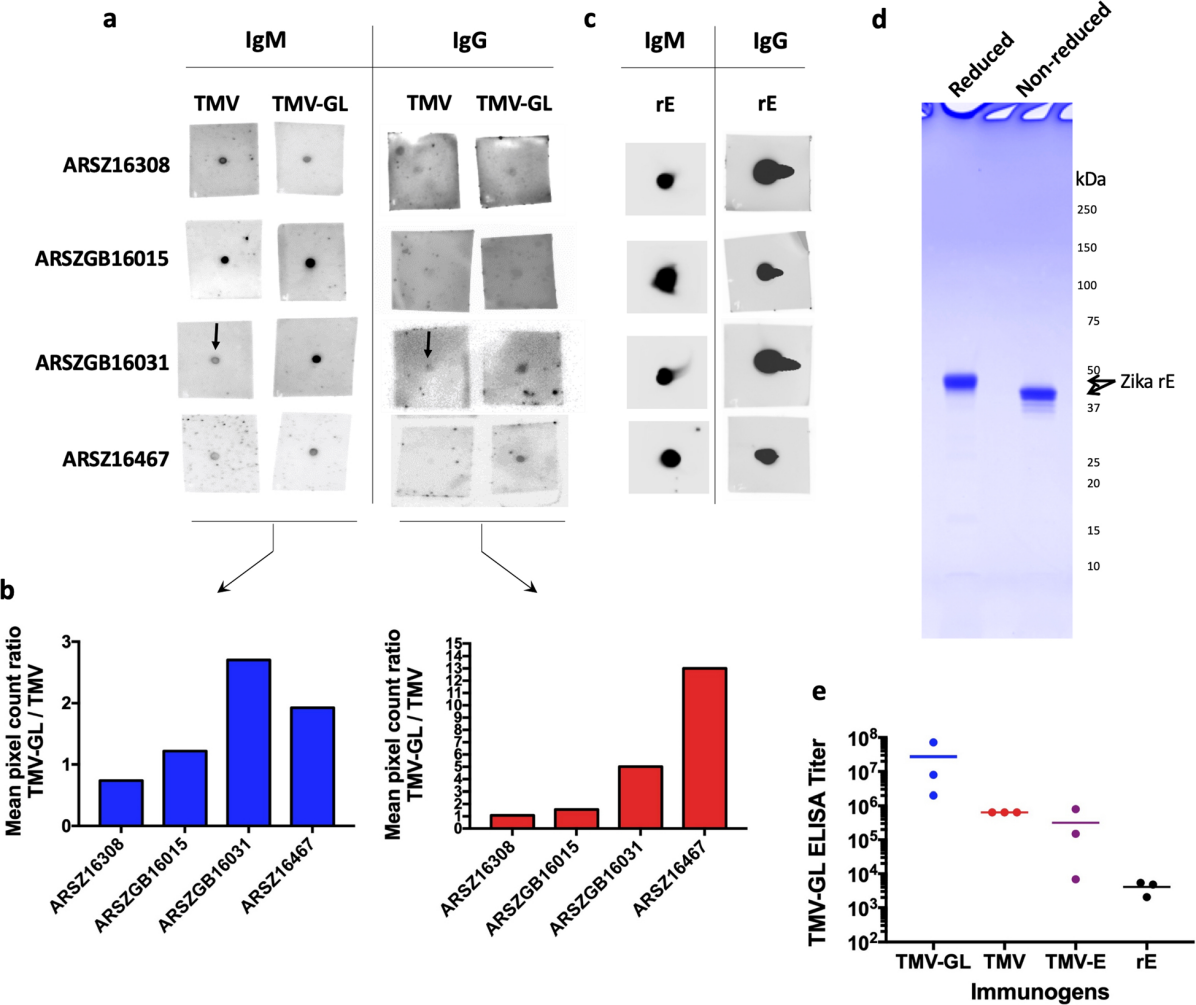
The immunogenicity of an unglycosylated glycan loop construct in humans and mice. **a** Dot blot analysis of human sera to detect IgM and IgG response to TMV-GL and TMV. Purified antigen (either TMV or TMV-GL) was spotted onto each square of nitrocellulose, dried, blocked, and then incubated with a 1:500 dilution of the indicated human serum in blocking buffer. After washing, the antibody bound to the membrane was detected using horseradish-peroxidase conjugated secondary antibodies and Clarity Western Substrate. **b** Digital quantification of the magnitude of dot blot assays using ImageJ software. The y-axis represents the mean pixel count ratio of the TMV-GL signal divided by the TMV signal. **c** Dot blot analysis of human sera to detect IgM and IgG responses to rE. Pooled sera from each of the 5 groups of immunized mice (TMV, TMV-GL, TMV-E, rE, and adjuvant only) were dotted onto nitrocellulose, blocked, and then incubated with a goat anti-mouse IgM HRP-conjugated secondary antibody and detection using Clarity Western Substrate. **d** Plant-expressed rE was run on a 4–20% SDS-PAGE gel and stained with Coomassie. rE was run either reduced or non-reduced, where reducing conditions break the disulfide bonds. Non-reducing conditions indicate that rE is not aggregated through inter-subunit disulfide bond, and rE runs as a monomer. **e** Endpoint ELISA titers against TMV-GL using sera from TMV-GL immunized mice, TMV immunized mice, TMV-E immunized mice, and rE immunized mice. Each point represents the endpoint titer of the sera from one mouse, and the bars indicate the average endpoint titer for each group. The difference between ELISA values generated by TMV-GL and TMV, TMV-E, or rE are statistically significant (*P* < 0.0001, F test)

All four patients exhibited clinical symptoms of ZIKV infection, ZIKV was serologically confirmed, and serum samples were collected 14–51 days after the onset of symptoms [44]. IgMs develop as an immediate response to viruses, but they typically disappear within 2–3 weeks of production, by which time IgGs have developed [45]. Since the serum samples were collected at different time points after the onset of symptoms, we tested for both IgM and IgG antibodies that recognize unglycosylated GL. Using dot blots, we determined that sera from patients ARSZGB16031 and ARSZ16467 appeared to contain IgM and IgG antibodies that bind to unglycosylated GL at levels higher than antibodies recognizing TMV (Figs. 5a and b). The presence of antibodies recognizing TMV could be due to prior exposure to TMV through contact with tobacco or tobacco products [46], exposure to non-tobacco plants containing TMV or a similar virus, or due to non-specific binding. Moreover, while all four serum samples appear to have similar levels of anti-rE IgM and IgG antibodies (Fig. 5c), GL-binding IgMs constitute a small but statistically significant portion of the total anti-rE IgM response, and IgGs recognizing unglycosylated GL constitute an even smaller fraction of total anti-E IgG Abs. Since these data show that natural ZIKV infections elicit GL-specific Abs in some patients, we investigated whether some of these Abs bind to monomeric or dimeric Es and whether those Abs are neutralizing.

### Unglycosylated GL is immunogenic in mice

While Wang et al. demonstrated that NAbs that recognize monomeric E and have molecular recognition sites within the GL region exist [10], we investigated whether we could find more of such Abs. Although soluble rE that lacks the transmembrane region has been shown to be monomeric under physiological conditions [7, 8, 10], rE monomers could, in principle, form multimers, especially during the purification and concentration process. In fact, it has been shown that Zika E protein may form disulfide bond-dependent aggregates in ZIKV-infected mammalian cells undergoing persistent endoplasmic reticulum stress [47]. To determine whether our plant-produced rE formed aggregates, we evaluated rE in reduced and non-reduced states on a SDS-PAGE gel (Fig. 5d), where reducing conditions would break any existing disulfide bonds. Under non-reducing conditions, we observed that rE was not aggregated through inter-subunit disulfide bonds and migrated as a monomer. In addition, to further maximize the likelihood of the monomeric state of rE, a TMV-E construct was produced where rE was displayed as a monomer on the surface of TMV. Immunocompetent female BALB/c mice were given intraperitoneal injections of 50 μg TMV, TMV-GL, rE, TMV-E in PBS with an adjuvant or PBS + adjuvant only. Mice were boosted approximately every three weeks thereafter, with a final boost was given 3 days prior to final bleeds and harvesting of spleens for cells fusions. The route of administration and the schedule of the immunization was comparable to previously published studies [48]. TMV-GL was used to determine if the unglycosylated GL was immunogenic. Glycosylated TMV-E and rE were included to ensure a robust immune response to Zika E, since a neutralizing immune response has previously been shown using plant-made rE [49]. TMV-E has the potential to further enhance the immune response by displaying rE on TMV using the Dock-And-Lock method. This method allows two proteins, rE and TMV coat protein in this case, to be purified separately and covalently attached in vitro. It is also possible that the glycan loop in TMV-GL may not elicit a neutralizing response due to suboptimal display on TMV but could be more antigenic in the context of the entire protein. Furthermore, glycosylated TMV-E and rE can be used to investigate whether any of the Abs generated in response to glycosylated rE are capable of binding to unglycosylated GL. In addition, one group of mice received wild-type TMV to control for and exclude any immune response to the scaffold virus.

We proceeded to study the IgG response, as these Abs provide long-term immunity. We measured TMV-GL specific IgG responses of individual mice by ELISA. Endpoint titers were defined as the reciprocal serum dilution, at which the absorbance at 450 nm (A450) was higher than the A450 signal of the pooled serum of the control mice (i.e., those injected with PBS + adjuvant) (Fig. 5e). We observed that mice immunized with TMV-GL produced the highest levels of IgG against TMV-GL. In addition, the immune response is higher to TMV-GL than to TMV alone, suggesting that the GL portion of TMV-GL is eliciting antibodies despite being such a small part of the VLP. Importantly, the serum of mice immunized with rE reacted with TMV-GL, which strongly suggests that the immune response to monomeric glycosylated E includes Abs that recognize GL independently of the glycan. Serum of mice immunized with either TMV-E or TMV reacted with TMV-GL, which could be from Abs that recognize either TMV or GL portions of the construct. This data shows that unglycosylated GL is immunogenic in mice, and that some of the Abs raised against monomeric glycosylated E recognize GL in a glycan-independent manner.

### Sera from immunized mice neutralizes ZIKV in vitro

**Fig. 6:**
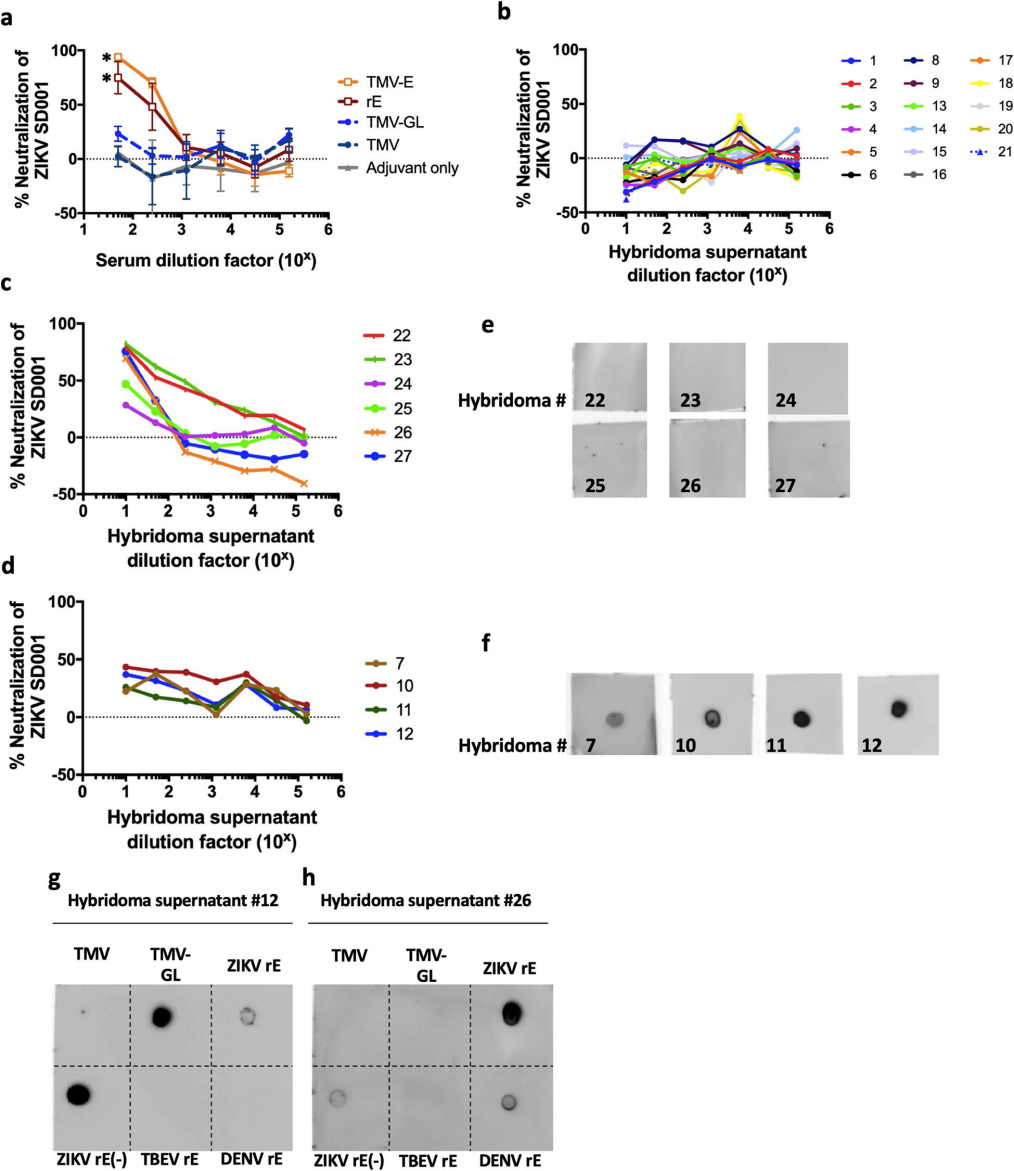
Neutralization of ZIKV by mouse serum and monoclonal antibody-containing hybridoma supernatants. a Individual serum samples from five groups of immunized mice (3 mice per group) were assessed for their ability to neutralize ZIKV (strain SD001) using a U937 DC-SIGN flow cytometry-based neutralization assay. The sera were serially diluted five-fold, starting at a 50x dilution. Each point represents the mean of percent neutralization, and the error bars depict the standard deviations. Asterisks indicate a statistical significance achieved at the lowest dilutions of TMV-E and rE samples as compared to adjuvant only sample (*P* < 0.0001, F test). **b**) Sub-neutralizing hybridoma supernatants. The ability of fourteen TMV-GL hybridoma supernatants, and two TMV-E supernatants were assessed using a flow-cytometry based neutralization assay. c) and d) . Hybridoma supernatants from **c**) an rE immunized mouse and **d**) a TMV-E immunized mouse were serially diluted five-fold starting at a 10x dilution. e) and f) Dot blot assays to detect hybridoma supernatants that bind to TMG-GL. TMV-GL was dotted onto nitrocellulose and probed with hybridoma supernatants from **e**) an rE immunized mouse or **f**) a TMV-E immunized mouse, and then detected using horseradish-peroxidase conjugated secondary antibodies and Clarity Western Substrate. g) and h) representative dot blots showing binding patterns of hybridoma supernatants. Hybridoma supernatant #12, from a TMV-E immunized mouse (**g**), and hybridoma supernatant #26, from an rE immunized mouse (**h**), were tested for their ability to bind to blotted TMV, TMV-GL, plant-made ZIKV rE (rE), *E. coli*-made ZIKV rE (rE(−)), TBEV rE, and DENV-2 rE. (−) indicates the lack of glycosylation

In order to investigate whether the sera from immunized mice were neutralizing, the pathogenicity of ZIKV strain SD001 in U937 + DC-SIGN cells was measured using flow-cytometry based neutralization assays. SD001 is a low passage (P2) clinical ZIKV isolate of Asian-lineage, which was isolated from a San Diegan who travelled to Venezuela in 2016 [50]. U937 cells, rather than Vero monkey kidney cells, were used because they are human monocytes, one of the physiological targets of ZIKV [51]. Moreover, unlike Vero cells, U937 cells can produce type I interferon and can respond to type I interferon signaling [50]. Flow-cytometry-based neutralization assays are routinely used to study Flaviviruses, and they provide comparable results to plaque reduction neutralization assays [52]. While sera from mice immunized with TMV-E or rE neutralized nearly 100% of viral propagation in the cellular assays at a 1:50 dilution (NT50s or the reciprocal serum dilution resulting in 50% neutralization were 560 and 235 for TMV-E and rE, respectively), sera from mice immunized with TMV, TMV-GL, or adjuvant alone did not contain significant neutralization activity (Fig. 6a). These data show that unglycosylated GL in the absence of other E regions is not sufficient to generate a neutralizing Ab response against ZIKV or that the unglycosylated GL in TMV-GL is not displayed to elicit a neutralizing response. As observed in a previous study with plant-made rE [49], these data also show that rE and TMV-E are sufficient to elicit a neutralizing response.

### ZIKV-neutralizing monoclonal antibodies bind to unglycosylated GL

Since the sera from TMV-GL immunized animals were not neutralizing, but rE and TMV-E immunized animal sera were neutralizing, we next focused on whether any of the NAbs from TMV-E and rE immunized mice were directed towards GL. Monoclonal antibodies (mAbs) were made from mice immunized with TMV-E and rE. Hybridomas were also produced from a TMV-GL immunized mouse, although they did not produce neutralizing antibodies (Fig. 6b). All hybridoma supernatants were screened for mAbs capable of binding to plant-produced glycosylated rE, and the 26 clones that bound to rE were cultured further (Table 3).

**Table 3:** Details of mouse hybridoma supernatants

Number	Hybridoma Supernatant Name	Mouse	Antigen
1	Z10C11–3–2-1-2	#287	TMV-GL
2	Z5B5–2–1-1-2	#287	TMV-GL
3	EV4E8–6–2-3-2	#300	TMV-E
4	Z10C11–3–2-3-2	#287	TMV-GL
5	Z9B12–2–2-2-1	#287	TMV-GL
6	Z9B12–2–2-1-1	#287	TMV-GL
7	EV4E8–5–4-3-1	#300	TMV-E
8	Z5B5–2–1-3-1	#287	TMV-GL
9	Z5B5–2–1-3-2	#287	TMV-EGL
10	EV4E8–5–1-1-1	#300	TMV-E
11	EV4E8–5–4-3-2	#300	TMV-E
12	EV4E8–5–1-1-3	#300	TMV-E
13	Z9B12–2–2-2-3	#287	TMV-GL
14	Z9B12–2–2-1-1	#287	TMV-GL
15	EV4E8–6–1-3-2	#300	TMV-E
16	Z10C11–3–2-3-1	#287	TMV-GL
17	Z5B5–2–1-1-1	#287	TMV-GL
18	Z9B12–2–2-1-4	#287	TMV-GL
19	Z9B12–2–2-2-2	#287	TMV-GL
20	Z9B12–2–2-1-3	#287	TMV-GL
22	KV14H8–1–2-1	#299	rE
23	KV14H8–1–2-2	#299	rE
24	KV13A7–2–3-2	#299	rE
25	KV13A7–2–3-1	#299	rE
26	KV13A7–2–6-2	#299	rE
27	KV13A7–2–6-1	#299	rE

As with the mouse sera, the ability of the mAb-containing hybridoma supernatants to neutralize ZIKV was examined using a flow-cytometry based neutralization assay. Four out of the six mAbs (clones #22, 23, 26, and 27) from an rE-immunized mouse strongly neutralized the virus (Fig. 6b and c), and two (clones #24 and 25) were moderately neutralizing. Moreover, four out of the six mAbs from a TMV-E immunized mouse moderately neutralized ZIKV (Fig. 6b and d). All ten mAbs were then analyzed via dot blot for their ability to bind to unglycosylated TMV-GL (Fig. 6e-f). All four of the moderately neutralizing mAbs from TMV-E bound to the unglycosylated GL (Fig. 4), while none of the neutralizing mAbs from rE bound to the GL (Fig. 6e). Additional immunoblotting showed that the GL-binding Abs, for example #12, did not recognize E of other flaviviruses, DENV-2 and TBEV, and showed preferential binding to unglycosylated rE expressed in *E. coli*, over plant-glycosylated rE (Fig. 6g). In contrast, mAb #26 cross-reacted with DENV-2 rE, and preferentially bound to plant-glycosylated ZIKV rE compared to unglycosylated rE expressed in *E. coli* (Fig. 6h).

## Discussion

The glycosylation site at N154 is a conserved feature of flaviviruses and has been shown to be important for infectivity and assembly [53–55], but the extended loop surrounding it is unique to neuroinvasive flaviviruses (i.e., WNV, JEV, and ZIKV). The extended ZIKV GL shares very little sequence identity with the WNV and JEV, making it a promising antigen for eliciting ZIKV-specific antibody response. Furthermore, computational approaches have predicted multiple B cell epitopes in the GL region [56, 57], and Xu et al. showed that the GL region of ZIKV has low sequence identity to other flaviviruses and high surface accessibility [56].

In this study, we immunized mice with rE and TMV-E and isolated mAbs that neutralize ZIKV, presumably by binding to E epitopes the infectious ZIKV particles. Furthermore, these NAbs either recognize unglycosylated GL, or require glycan for their efficient binding to monomeric E. Based on the existing nomenclature of EDE mAbs, we propose a new classification for Abs that bind to E monomer epitopes (EME): EME1 for antibodies that bind better to E monomers without glycosylation at N154 and EME2 for those that require an N154 glycan to bind to E.

mAbs that bind to E neutralize ZIKV by blocking attachment factors, obstructing binding to entry receptors, or hindering the membrane fusion process. Many cellular receptors have been implicated in ZIKV binding and entry, including AXL, Tryo3, DC-SIGN, and TIM-1 [58], and any glycans present on E could play a role in viral entry [12, 13]. The fact that the N154 glycosylation site remains highly conserved among flaviviruses suggests it has an important function. However, ZIKV particles lacking the E glycan were capable of infecting Raji cells expressing the lectin DC-SIGN, so the prM glycan on partially mature particles may be able to facilitate entry as well [20]. Additional studies demonstrate that ZIKV utilizes different host cell receptors in different tissues [59], and each receptor could bind to a different part of the E and/or prM. Consequently, an antibody that blocks a single binding site may only neutralize ZIKV in specific tissues, and thus may only have limited therapeutic potential. EDE-binding mAbs tend to neutralize by locking the E dimers, which prevents membrane fusion. By their nature, EME mAbs cannot lock E dimers, but they may still impede the fusion process by sterically hindering the change from the dimeric form to the trimeric form during membrane fusion. GL has been shown to at least partially cover the FL on ZIKV [5] and blocks neutralization by FL-binding mAbs [20], so EME1 could hinder fusion by blocking FL and preventing membrane fusion. The true mechanism by which the NAbs we describe here function to neutralize the Zika virus is not yet known.

## Conclusions

Recent studies have shown that the glycan loop (GL) of the ZIKV envelope protein (E) plays an important role in the virus antigenicity and pathogenesis. To better elucidate the role the GL plays in the ZIKV immune response, we immunized mice with GL or full-length monomeric E and analyzed their immune response. While GL alone was not sufficient to induce a neutralizing immune response, this work demonstrates that this region does play an important role that has been largely overlooked. The inclusion of monomeric E in the immunization study led to the discovery of at least two moderately neutralizing monoclonal antibodies. The novelty of antibodies that bind to E monomer epitopes lies in their ability to bind to a unique GL region of the ZIKV E that has little homology with other flaviviruses. Although further studies are needed, it is likely that such antibodies will not bind to other flaviviruses, which may prove advantageous for both therapeutic and diagnostic applications.

## Data Availability

Not applicable.

## Abbreviations

ZIKV: Zika virus
TMV: Tobacco mosaic virus
NAb: Neutralizing antibody
mAb: Monoclonal antibody
E: Envelope
rE: Recombinant envelope
GL: Glycan loop
EDE: E dimer epitopes
EME: E monomer epitopes (EDE)
